# Immunity against measles, mumps, rubella, and varicella among homeless individuals in Germany — A nationwide multi-center cross-sectional study

**DOI:** 10.3389/fpubh.2024.1375151

**Published:** 2024-05-09

**Authors:** Wiebke Graf, Franziska Bertram, Katharina Dost, Anna Brennecke, Veronika Kowalski, Victoria van Rüth, Dominik Sebastian Nörz, Birgit Wulff, Benjamin Ondruschka, Klaus Püschel, Susanne Pfefferle, Marc Lütgehetmann, Fabian Heinrich

**Affiliations:** ^1^Institute of Legal Medicine, University Medical Center Hamburg-Eppendorf, Hamburg, Germany; ^2^Institute of Medical Microbiology, Virology and Hygiene, University Medical Center Hamburg-Eppendorf, Hamburg, Germany; ^3^Department of Medical Statistics, London School of Hygiene and Tropical Medicine, London, United Kingdom; ^4^Center for Data and Statistical Science for Health, London School of Hygiene and Tropical Medicine, London, United Kingdom

**Keywords:** MMR, MMRV, homelessness, homeless, seroprevalence, immune protection, immunity

## Abstract

**Introduction:**

Homeless individuals suffer a high burden of vaccine-preventable infectious diseases. Moreover, they are particularly susceptible to adverse infection outcomes with limited access to the health care system. Data on the seroprevalence of measles, mumps, rubella, and varicella within this cohort are missing.

**Methods:**

The seroprevalence of measles, mumps, rubella, and varicella was determined within the homeless population in Germany. Predictors of lacking immune protection were determined using multivariable logistic regression analysis.

**Results:**

Homeless individuals in Germany (*n* = 611) showed a seroprevalence of 88.5% (95% CI: 85.8–91.0) for measles, 83.8% (95% CI: 80.6–86.6) for mumps, 86.1% (95% CI: 83.1–88.7) for rubella, and 95.7% (95% CI 93.8–97.2) for varicella. Measles seroprevalences declined from individuals born in 1965 to individuals born in 1993, with seroprevalences not compatible with a 95% threshold in individuals born after 1980. For mumps, seroprevalences declined from individuals born in 1950 to individuals born in 1984. Here, seroprevalences were not compatible with a 92% threshold for individuals born after 1975. Seronegativity for measles, mumps and rubella was associated with age but not with gender or country of origin.

**Discussion:**

Herd immunity for measles and mumps is not achieved in this homeless cohort, while there was sufficient immune protection for rubella and varicella. Declining immune protection rates in younger individuals warrant immunization campaigns also targeting marginalized groups such as homeless individuals. Given that herd immunity thresholds are not reached for individuals born after 1980 for measles, and after 1975 for mumps, vaccination campaigns should prioritize individuals within these age groups.

## 1 Introduction

Approximately 417,000 homeless individuals were living in Germany in 2020 (1). Homeless individuals are exposed to various non-communicable and communicable diseases, including vaccine-preventable diseases (2–4). Poor and overcrowded living conditions, limited access to sanitary care, and reduced hygienic standards facilitate their spread in this cohort (5). This is especially true for droplet and aerogenic transmitted diseases. As well as this, high mobility in this cohort impedes the control of the spread of such diseases, thus making homeless shelters high-risk sites for infectious disease outbreaks (2, 3, 6). High rates of somatic and psychiatric diseases in combination with high rates of risk behaviors such as alcohol consumption, tobacco usage, substance abuse, and victimization make the homeless population especially vulnerable (7–12). In line with that, studies reveal a significant reduction in the life expectancy of homeless individuals (13), associated with an overrepresentation of infectious diseases as a potential underlying cause of death (14). Furthermore, a lack of health insurance, trust in health care providers, and feeling unwelcome are known barriers to active participation in the general health care system (15–17).

Measles, mumps, rubella, and varicella (MMRV) are highly contagious viral diseases that typically occur during childhood and adolescence and are transmitted via droplet or aerogenic infection. However, infection is also possible in unprotected adults (18). MMRV Infection can cause varying disease severities - from mild to severe - yet can simultaneously cause serious complications (19–22). Measles cases have fluctuated in Germany and other European countries in recent years. While 545 measles cases have been reported to the German National Public Health Institute (Robert Koch Institute, RKI) in 2018, only 15 cases have been reported in 2022. Similarly, around 18,000 measles cases were reported in European countries in 2018 while only 127 cases were reported in 2022. This can be mainly attributed to control measures during the COVID-19 pandemic (23, 24). However, since the end of the COVID-19 pandemic, the number of measles cases has alarmingly increased again. In Germany around 80 cases were reported in 2023 while 37 cases were reported in January 2024 alone (25). The World Health Organization (WHO) similarly reported a surge in measles cases in European countries following the end of the COVID-19 pandemic in 2023, which is, among others, attributed to declining vaccination rates from 2020 to 2022 except for SARS-CoV-2 (26).

The number of acute rubella cases in Germany has been constantly low since 2020, when the WHO granted rubella it's elimination status. Only eight cases of congenital rubella were reported to the RKI in 2022 for Germany (27). Likewise, low numbers of rubella cases are reported in most European countries. In an annual epidemiological report by the ECDC from 2017, about 700 rubella cases were reported in European countries, with Poland, Germany, Italy, and Austria reporting the highest number of cases (28).

Since 2013, an average of 700 cases of mumps have been reported in Germany every year. More specifically, while there were reported around 900 cases in 2017, the number declined to around 250 in 2021 and increased again with around 600 cases reported in 2023 (29). For mumps cases in Europe, the ECDC reported a decline from around 11000 in 2018 to 1600 in 2021, with most cases reported in Spain, Italy, Poland, and the United Kingdom. Furthermore, several mumps outbreaks have been recorded across Germany and beyond, constituting the disease burden attributed to mumps in recent decades (30).

In Germany varicella cases also fluctuated in recent years. While 20,500 cases have been reported in 2018, 6,400 and 18,000 cases were reported in 2021 and 2023, respectively, potentially reflecting the influence of the COVID-19 pandemic (29). Unfortunately, no surveillance system exists for varicella cases across European countries. An annual report from the ECDC in 2010 reported around 600,000 varicella cases in 18 European countries (31).

In Germany, vaccines against measles, mumps and rubella were first recommended by the German Standing Vaccination Committee (STIKO) in the 1970s. The measles-mumps-rubella (MMR) combination vaccine has been widely used since the 1980s. It was not until 2004 that vaccination against varicella was recommended by the STIKO also (32). Since 2006, the MMRV combination vaccine has been widely available in Germany (33). Currently, the STIKO recommends two shots with an MMR(V) combination vaccine, with the first dosage being administered at 11 months and the second at 15 months of age. Regarding measles, individuals born after 1970 without complete vaccination or an unclear vaccination or infection status should receive a single shot with an MMR combination vaccine (34). All individuals born after 1970 who are cared for in a community facility or work in medical and community settings must prove measles protection (35). While no specific recommendation exists for homeless individuals, asylum seekers and refugees require vaccination within 4 weeks of admission to a shared accommodation (36). Overall, European countries generally recommend MMR vaccination, with two dosages administered during childhood. MMR vaccination is even mandatory in some countries, such as France and Italy. In contrast, vaccine recommendations regarding varicella are more heterogeneous; in some European countries, it is not recommended or not funded by the national health system, while others mandate it (37–40).

Vaccination data of the general population are not systematically collected. Only data from school entry health examinations and systematic evaluations of healthcare providers are available to estimate the latest vaccination rates. However, current data on the immunization status of a population are necessary to identify populations at risk and, thus, generate targeted vaccination recommendations and related campaigns (41). Recent data from the German Health Interview and Examination Survey (DEGS1) showed high seroprevalence for measles, mumps and rubella, respectively, in the German general adult population (42).

No representative data on the seroprevalence of MMRV in homeless adults in Germany is available, while recent studies suggest that more than 60% of homeless individuals in the United States were unsure about their vaccination status (43).

Having limited access to the healthcare system, homeless individuals might not be sufficiently reached through vaccination programs while at the same time having a significant need for vaccine protection. This study aimed to investigate the seroprevalence of measles, mumps, rubella, and varicella antibodies in a representative sample of individuals experiencing homelessness in Germany. Moreover, we aimed to identify key predictors of lacking humoral immune protection to identify subgroups at risk of infection.

## 2 Materials and methods

### 2.1 Study design and data collection

Data were collected between July and September 2021 as part of the National Survey on the Psychiatric and Somatic Health of Homeless Individuals (NAPSHI) in four German metropolitan areas around Hamburg, Frankfurt, Leipzig, and Munich. Before starting the enrolment process, study municipal authorities in the Hamburg, Frankfurt, Leipzig, and Munich areas were contacted. Contact information was requested from institutional representatives of public spaces, shelters, lodging houses, drug aid facilities, women's shelters, and medical practices offering specialized care for individuals experiencing homelessness. The authorities then provided contact information for all sites willing to participate. In total, 39 homeless support facilities all over Germany participated in the survey. Sites were contacted in advance, and information on the study and material for study advertisement was sent to these facilities at least 2 weeks before enrollment. The study team of medical doctors and students visited each site. Every individual on-site was approached for participation in the study. Overall, 699 participants were recruited. Individuals were included when accommodated according to operational categories 1 to 5 and 7 in the European Typology of Homelessness and Housing Exclusion (ETHOS). ETHOS classifies homeless people according to their housing situation. We included homeless individuals living rough (category 1), in emergency accommodations (category 2), in accommodation for the homeless (category 3), in women's shelters (category 4), in accommodation for immigrants (category 5), and long-term accommodation (category 7). In our sample, no homeless individual reported recent release from institutions (category 6) (44). A more detailed description can be found in Bertram et al. (45).

Inclusion criteria were the lack of permanent residence (> 7 days) and age > 18 years. Pregnant individuals were not included in the study. Written informed consent was obtained and documented. An allowance of 5.00€ per 0.5 h was offered. Data on demographics and psychiatric and somatic illnesses were collected via questionnaires. When possible, questionnaires were filled out by the participants independently. However, most participants were interviewed face-to-face to overcome difficulties in reading or understanding the questions. In addition, a venous blood withdrawal was conducted by trained medical professionals. Serum blood samples were centrifuged, stored, and transported to the University Medical Center Hamburg-Eppendorf at 4°C. Samples were analyzed at the Institute of Medical Microbiology, Virology, and Hygiene. One individual was excluded because they did not fulfill the inclusion criteria and 27 were excluded because inclusion criteria were not available. Of 671 individuals eligible for study inclusion, 60 individuals were excluded from the primary analysis due to missing blood withdrawals (see Supplementary Table 1). The final analytic sample was composed of 611 homeless individuals. The study was conducted in accordance with the declaration of Helsinki. Ethical approval was obtained from the ethics committee of the Hamburg Chamber of Physicians (application number: PV7333).

### 2.2 Sociodemographic data

Basic sociodemographic variables were evaluated, i.e., gender (male, female), age (in years), country of origin (Germany, European (EU), non-EU), level of education (no degree, school education, vocational education, higher tertiary education), marital status (single, married, divorced, widowed), and occupational status (yes, no). Additionally, we included information on the homeless access to social security systems, i.e., availability of health insurance (no, yes) and availability of welfare (yes, no). Social welfare includes financial support for costs of living, accommodation as well as special needs such as illness and disability for individuals in need who are unable to provide for themselves.

### 2.3 Serological assays

Anti-measles IgG level (quantitative assay range 5-300 AU/mL), anti-mumps IgG level (quantitative assay range 5-300 AU/mL), and anti-VZV IgG level (quantitative assay range 10-4000 mIU/mL) were determined by chemiluminescence immunoassay (CLIA) using an automated analyser (Liasion XL, Diasorine, Italy). Anti-rubella IgG levels (quantitative assay range 0-500 IU/mL) were determined using the Alinity I system (Abbott, Wiesbaden, Germany). Measurements were performed in an accredited virology laboratory of the University Medical Center Hamburg-Eppendorf according to the respective manufacturer's recommendation. Measles virus IgG levels equal to or above 16.5 AU/mL were considered seropositive, between 13.5 and 16.5 AU/mL were considered equivocal, and below 13.5 AU/mL were considered seronegative. Rubella virus IgG levels ≥ 10.0 IU/mL were considered seropositive, between 5.0 to 9.9 IU/mL were considered equivocal, and 0.0 to 4.9 IU/mL were considered seronegative. Mumps virus IgG levels above 11.0 AU/mL were considered seropositive, between 9.0 and 11.0 AU/mL were considered equivocal, and below 9.0 AU/mL were considered seronegative. Varicella virus IgG levels ≥ 100 mIE/mL were considered seropositive, between 50 and 100 mIE/mL were considered equivocal, and below 50 mIE/mL were considered seronegative. All assays were calibrated using international standards. The measles IgG cut-off value for seropositivity equals 175 mIU/mL (WHO Third International Standard for Anti-Measles, NIBSC code 97/648). VZV IgG mIU is calibrated against WHO International Preparation W1044. Rubella IgG cut-off value for seropositivity was 10 IU/mL (calibrated against the WHO 1st International Standard for Anti-Rubella Immunoglobulin).

### 2.4 Statistical analysis

Categorical variables are presented as numbers and percentages. Continuous variables were presented as mean and SD, or median and IQR as appropriate. Exact 95% confidence intervals were calculated from the binomial distribution. A generalized linear model with binomial family and logit link was used to identify determinants of negative seroprevalence for measles, mumps, rubella, and varicella. Equivocal levels were defined as negative. Serostatus (0/1) served as the dependent variable; independent variables were included on a clinical basis. Age (continuous), gender (categorical), and country of origin (categorical) were included as independent variables. Potential model misspecification was assessed using the Hosmer-Lemeshow test with 10 groups. Standardized Pearson residuals were calculated. Index plots were used to assess for potentially influential values. Plots of the continuous variable in the model against standardized Pearson's residuals were used to examine the appropriate functional form of continuous variables. Statistical analysis was performed using Stata 17.0 (Stata Corp, College Station, TX, USA). The significance level was set at α = 0.05 for all statistical tests, indicating a 5% risk of Type I error when rejecting the null hypothesis.

## 3 Results

### 3.1 Sample characteristics

A detailed description of the sociodemographic data of the analyzed homeless cohort is shown in Table 1. The sample was composed of 611 homeless individuals. The median age was 43 years (IQR 35–53 years), and 83.4% (*n* = 509) of the individuals were male. A slight majority of the individuals, 51.4% (*n* = 301), were born in Germany. Of the homeless individuals, 192/611 (32.8%) immigrated from EU countries and 93/611 (15.9%) from non-EU countries. The median duration of homelessness was 18 months (IQR 6–48 months).

**Table 1:** Sample characteristics stratified by year of birth (*n* = 611).

	**Year of birth**												
	**1940–1944**	**1945–1949**	**1950–1954**	**1955–1959**	**1960–1964**	**1965–1969**	**1970–1974**	**1975–1979**	**1980–1984**	**1985–1993**	**1994–1999**	**2000–2004**	**Total**
	***n** =* **4**	***n** =* **4**	***n** =* **12**	***n** =* **19**	***n** =* **49**	***n** =* **84**	***n** =* **76**	***n** =* **84**	***n** =* **99**	***n** =* **118**	***n** =* **44**	***n** =* **18**	***n** =* **611**
**Independent variables**	**Median (IQR) /** ***n*** **(%)**												
**Gender**													
Male	3 (75.0%)	4 (100.0%)	10 (90.9%)	17 (89.5%)	40 (81.6%)	71 (84.5%)	64 (84.2%)	67 (79.8%)	80 (80.8%)	100 (84.7%)	39 (88.6%)	14 (77.8%)	509 (83.4%)
Female	1 (25.0%)	0 (0.0%)	1 (9.1%)	2 (10.5%)	9 (18.4%)	13 (15.5%)	12 (15.8%)	17 (20.2%)	19 (19.2%)	18 (15.3%)	5 (11.4%)	4 (22.2%)	101 (16.6%)
**Age (years)**	**79.5 (78.5–80)**	**74 (73.5–75)**	**69 (67.5–71)**	**64 (62–65)**	**59 (58–60)**	**54 (53–55)**	**49 (48–50)**	**44 (42–45)**	**39 (38–40)**	**33 (31–35)**	**24 (23–26)**	**20 (19–21)**	**43 (35–53)**
**Country of origin**													
Germany	3 (75.0%)	0 (0.0%)	7 (63.6%)	7 (38.9%)	29 (63.0%)	40 (48.8%)	40 (54.1%)	42 (51.9%)	46 (49.5%)	58 (50.4%)	20 (48.8%)	9 (50.0%)	301(51.4%)
EU-country	0 (0.0%)	1 (33.3%)	3 (27.3%)	7 (38.9%)	10 (21.7%)	32 (39.0%)	24 (32.4%)	32 (39.5%)	33 (35.5%)	36 (31.3%)	11 (26.8%)	3 (16.7%)	192 (32.8%)
Non-EU-country	1 (25.0%)	2 (66.7%)	1 (9.1%)	4 (22.2%)	7 (15.2%)	10 (12.2%)	10 (13.5%)	7 (8.6%)	14 (15.1%)	21 (18.3%)	10 (24.4%)	6 (33.3%)	93 (15.9%)
**Education**													
No degree	0 (0.0%)	3 (100.0%)	2 (18.2%)	1 (5.3%)	5 (10.4%)	8 (9.9%)	13 (18.1%)	9 (11.7%)	26 (26.8%)	22 (19.5%)	11 (25.6%)	6 (35.3%)	106 (18.1%)
School education	0 (0.0%)	0 (0.0%)	3 (27.3%)	10 (52.6%)	19 (39.6%)	33 (40.7%)	30 (41.7%)	38 (49.4%)	44 (45.4%)	57 (50.4%)	28 (65.1%)	10 (58.8%)	272 (46.5%)
Vocational education	3 (75.0%)	0 (0.0%)	3 (27.3%)	5 (26.3%)	18 (37.5%)	33 (40.7%)	23 (31.9%)	24 (31.2%)	22 (22.7%)	29 (25.7%)	2 (4.7%)	1 (5.9%)	163 (27.9%)
Higher tertiary education	1 (25.0%)	0 (0.0%)	3 (27.3%)	3 (15.8%)	6 (12.5%)	7 (8.6%)	6 (8.3%)	6 (7.8%)	5 (5.2%)	5 (4.4%)	2 (4.7%)	0 (0.0%)	44 (7.5%)
**Marital status**													
Married and married living apart	1 (25.0%)	2 (50.0%)	2 (16.7%)	1 (5.6%)	5 (10.4%)	14 (17.1%)	10 (13.9%)	10 (12.2%)	10 (10.4%)	12 (10.5%)	2 (4.9%)	0 (0.0%)	69 (11.8%)
Single	1 (25.0%)	1 (25.0%)	5 (41.7%)	9 (50.0%)	23 (47.9%)	47 (57.3%)	42 (58.3%)	54 (65.9%)	68 (70.8%)	90 (78.9%)	39 (95.1%)	15 (100.0%)	394 (67.0%)
Widowed	1 (25.0%)	0 (0.0%)	2 (16.7%)	0 (0.0%)	2 (4.2%)	2 (2.4%)	3 (4.2%)	2 (2.4%)	2 (2.1%)	1 (0.9%)	0 (0.0%)	0 (0.0%)	15 (2.6%)
Divorced	1 (25.0%)	1 (25.0%)	3 (25.0%)	8 (44.4%)	18 (37.5%)	19 (23.2%)	17 (23.6%)	16 (19.5%)	16 (16.7%)	11 (9.6%)	0 (0.0%)	0 (0.0%)	110 (18.7%)
**Occupation**	**0 (0.0%)**	**0 (0.0%)**	**2 (16.7%)**	**1 (5.6%)**	**7 (16.7%)**	**11 (14.3%)**	**11 (15.9%)**	**7 (9.1%)**	**10 (11.0%)**	**12 (10.5%)**	**5 (11.9%)**	**0 (0.0%)**	**66 (11.6%)**
**On welfare**	**0 (0.0%)**	**2 (50.0%)**	**4 (33.3%)**	**8 (42.1%)**	**24 (53.3%)**	**38 (46.3%)**	**34 (45.9%)**	**35 (43.2%)**	**47 (50.0%)**	**49 (42.2%)**	**20 (48.8%)**	**5 (27.8%)**	**266 (45.2%)**
**Health Insurance**	**3 (75.0%)**	**1 (25.0%)**	**10 (83.3%)**	**13 (68.4%)**	**37 (77.1%)**	**53 (63.9%)**	**51 (69.9%)**	**50 (60.2%)**	**66 (67.3%)**	**76 (66.1%)**	**29 (67.4%)**	**12 (75.0%)**	**401 (67.1%)**
**Duration of homelessness (months)**	**48 (18.4–132)**	**36 (18.5–90)**	**11 (2–36)**	**19 (10–54)**	**36 (12–96)**	**24 (7–90)**	**18 (5–48)**	**24 (6–60)**	**24 (6–60)**	**12 (6–32)**	**12 (4–24)**	**3 (3–8)**	**18 (6–48)**
**ETHOS**													
Living rough	0 (0.0%)	0 (0.0%)	6 (50.0%)	8 (44.4%)	17 (36.2%)	33 (41.3%)	30 (42.3%)	35 (45.5%)	43 (47.3%)	45 (40.2%)	13 (31.0%)	8 (44.4%)	238 (41.3%)
Emergency accomodation	1 (25.0%)	0 (0.0%)	2 (16.7%)	3 (16.7%)	9 (19.1%)	8 (10.0%)	6 (8.5%)	14 (18.2%)	11 (12.1%)	22 (19.6%)	11 (26.2%)	5 (27.8%)	92 (16.0%)
Accomodation for the homeless	3 (75.0%)	3 (75.0%)	3 (25.0%)	7 (38.9%)	15 (31.9%)	33 (41.3%)	27 (38.0%)	26 (33.8%)	31 (34.1%)	41 (36.6%)	16 (38.1%)	5 (27.8%)	210 (36.5%)
Womens shelter	0 (0.0%)	1 (25.0%)	1 (8.3%)	0 (0.0%)	5 (10.6%)	4 (5.0%)	7 (9.9%)	2 (2.6%)	4 (4.4%)	4 (3.6%)	2 (4.8%)	0 (0.0%)	30 (5.2%)
Accomodation for immigrants	0 (0.0%)	0 (0.0%)	0 (0.0%)	0 (0.0%)	1 (2.1%)	1 (1.3%)	1 (1.4%)	0 (0.0%)	0 (0.0%)	0 (0.0%)	0 (0.0%)	0 (0.0%)	3 (0.5%)
People in long term accommodation	0 (0.0%)	0 (0.0%)	0 (0.0%)	0 (0.0%)	0 (0.0%)	1 (1.3%)	0 (0.0%)	0 (0.0%)	2 (2.2%)	0 (0.0%)	0 (0.0%)	0 (0.0%)	3 (0.5%)

Numbers and percentages of non-missing observations are depicted. EU, European; ETHOS, European Typology of Homelessness.

### 3.2 Seroprevalence of MMRV

The seroprevalence of MMRV in the homeless population in Germany is shown in Table 2. The overall prevalence was 88.5% (95% CI: 85.8–91.0) for anti-measles-IgG, 83.8% (95% CI: 80.6–86.6) for anti-mumps-IgG, 86.1 % (95% CI 83.1–88.7) for anti-rubella-IgG, and 95.7% (95% CI: 93.8–97.2) for anti-varicella-IgG. Seroconversion and thus estimated immune protection against all three (MMR) and all four (MMRV) diseases was shown in 68.0% (95% CI: 64.1–71.6) and 66.0% (95% CI: 61.7–69.4) of homeless individuals, respectively.

**Table 2:** Seroprevalence of measles, mumps, rubella, and varicella in the homeless population in Germany stratified by gender and year of birth (*n* = 611 for measles, mumps, rubella, and varicella).

	**Measles level**			**Mumps level**			**Rubella level**			**Varicella level**		
**Seroprevalence %**	**Sero-negative %**	**Equivocal %**	**Sero-positive %**	**Sero-negative %**	**Equivocal %**	**Sero-positive %**	**Sero-negative %**	**Equivocal %**	**Sero-positive %**	**Sero-negative %**	**Equivocal %**	**Sero-positive %**
**(95% CI)**												
**Total**	10.8 (8.5–13.5)	0.7 (0.2–1.7)	88.5 (85.8–91.0)	15.2 (12.5–18.3)	1.0 (0.4–2.1)	83.8 (80.6–86.6)	9.8 (7.6–12.5)	4.1 (2.7–6.0)	86.1 (83.1–88.7)	3.3 (2.0–5.0)	1.0 (0.4–2.1)	95.7 (93.8–97.2)
**Gender**												
Men	11.2 (8.6–14.3)	0.8 (0.2–2.0)	88.0 (84.9–90.7)	15.7 (12.7–19.2)	0.8 (0.2–2.0)	83.5 (80.0–86.6)	10.8 (8.2–13.8)	3.7 (2.3–5.8)	85.5 (82.1–88.4)	3.1 (1.8–5.1)	0.8 (0.2–2.0)	96.1 (94.0–97.6)
Women	8.9 (4.2–16.2)	0.0 (0.0–0.0)	91.1 (83.8–95.8)	12.9 (7.0–21.0)	2.0 (0.2–7.0)	85.2 (76.7–91.4)	5.0 (1.6–11.2)	5.9 (2.2–12.5)	89.1 (81.4–94.4)	4.0 (1.1–9.8)	2.0 (0.2–7.0)	94.1 (87.5–97.8)
**Year of birth**												
1940–1944	0.0 (0.0–60.2)	0.0 (0.0–60.2)	100.0 (39.8–100.0)	25.0 (0.6–80.6)	0.0 (0.0–60.2)	75.0 (19.4-99.37)	0.0 (0.0–60.2)	0.0 (0.0–60.2)	100.0 (39.8-100.0)	0.0 (0.0–60.2)	0.0 (0.0–60.2)	100.0 (39.8–100.0)
1945–1949	0.0 (0.0–60.2)	0.0 (0.0–60.2)	100.0 (39.8–100.0)	0.0 (0.0–60.2)	0.0 (0.0–60.2)	100.0 (39.8-100.0)	0.0 (0.0–60.2)	0.0 (0.0–60.2)	100.0 (39.8–100.0)	0.0 (0.0–60.2)	0.0 (0.0–60.2)	100.0 (39.8–100.0)
1950–1954	0.0 (0.0–26.5)	0.0 (0.0–26.5)	100.0 (73.5–100.0)	8.3 (0.2–38.5)	0.0 (0.0–26.5)	91.7 (61.5-99.8)	0.0 (0.0–26.5)	0.0 (0.0–26.5)	100.0 (73.5–100.0)	0.0 (0.0–26.5)	0.0 (0.0–26.5)	100.0 (73.5–100.0)
1955–1959	0.0 (0.0–17.6)	0.0 (0.0–17.6)	100.0 (82.4–100.0)	5.3 (0.1–26.0)	0.0 (0.0–17.6)	94.7 (74.0-99.9)	5.3 (0.1–26.0)	5.3 (0.1–26.0)	89.5 (66.9-98.7)	5.3 (0.1–26.0)	0.0 (0.0–17.6)	94.7 (74.0–99.9)
1960–1964	0.0 (0.0–7.3)	0.0 (0.0–7.3)	100.0 (92.8–100.0)	12.2 (4.6–24.8)	0.0 (0.0–7.3)	87.8 (75.2–95.4)	10.2 (3.4–22.2)	4.1 (0.5–14.0)	85.7 (72.8–94.1)	6.1 (1.3–16.9)	0.0 (0.0–7.3)	93.9 (83.1–98.7)
1965–1969	2.4 (0.3–8.3)	0.0 (0.0–4.3)	97.6 (91.6-99.7)	7.1 (2.7–14.9)	1.2 (0.0–6.5)	91.7 (83.6–96.6)	7.1 (2.7–14.9)	0.0 (0.0–4.3)	92.9 (85.1–97.3)	1.2 (0.0–6.5)	0.0 (0.0–4.3)	98.8 (93.5–100.0)
1970–1974	6.6 (2.2–14.7)	0.0 (0.0–4.7)	93.4 (85.3–97.8)	7.9 (3.0–16.4)	1.3 (0.0–7.1)	90.8 (81.9–96.2)	6.6 (2.1–14.7)	2.6 (0.3–9.2)	90.8 (81.9–96.2)	0.0 (0.0–4.7)	1.3 (0.0–7.1)	98.7 (92.9–100.0)
1975–1979	9.5 (4.2–17.9)	0.0 (0.0–4.3)	90.5 (82.1–95.8)	16.7 (9.4–26.4)	0.0 (0.0–4.3)	83.3 (73.6–90.6)	11.9 (5.9–20.8)	2.4 (0.0–5.6)	85.7 (76.4–92.4)	6.0 (2.0–13.4)	0.0 (0.0–4.3)	94.1 (86.7–98.0)
1980–1984	17.2 (10.3–26.1)	1.0 (0.0–5.5)	81.8 (72.8–88.9)	24.2 (16.2–33.9)	1.0 (0.0–5.5)	74.8 (65.0–82.9)	14.1 (8.0–22.6)	3.0 (0.3–8.3)	82.8 (73.9–89.7)	3.0 (0.6–8.6)	2.0 (0.3–7.1)	95.0 (88.6–98.3)
1985–1993	23.7 (16.4–32.4)	0.9 (0.0–4.6)	75.4 (66.7–82.9)	22.0 (14.9–30.6)	0.9 (0.0–4.6)	77.1 (68.5–84.4)	12.7 (7.3–20.1)	8.5 (4.1–15.0)	78.8 (70.3–85.8)	4.2 (1.4–9.6)	2.5 (0.5–7.3)	93.2 (87.1–97.0)
1994–1999	9.1 (2.5–21.7)	4.6 (0.6–15.5)	86.4 (72.7–94.8)	13.6 (5.2–27.4)	2.3 (0.1–12.0)	84.1 (69.9–93.4)	2.3 (0.1–12.0)	6.8 (1.4–18.7)	90.9 (78.3–97.5)	2.3 (0.1–12.0)	0.0 (0.0–8.0)	97.7 (88.0–99.9)
2000–2004	11.1 (1.4–34.7)	0.0 (0.0–18.5)	88.9 (65.3-98.6)	11.1 (1.4–34.7)	5.6 (0.1–27.3)	83.3 (58.6-96.4)	16.7 (3.6–41.4)	11.1 (1.4–34.7)	72.2 (46.5–90.3)	5.6 (0.1–27.3)	0.0 (0.0–18.5)	94.4 (72.7–100.0)

CI, confidence interval.

#### 3.2.1 Seroprevalence of measles in the homeless population

Examining the seroprevalence of measles in the homeless population in Germany, we found 541/611 [88.5% (95% CI: 85.7–90.9)] homeless individuals to be seropositive. 4/611 [0.7% (95% CI: 0.2–1.7)] displayed an equivocal level of IgG antibodies, while 66/611 [10.8% (95% CI: 8.5–13.6)] were seronegative. In detail, 57/509 [11.2% (95% CI: 8.6–14.3)] of men were seronegative, while 9/101 [8.9% (95% CI: 4.2–16.2)] of women were seronegative (Table 2). Overall, the seropositivity proportion stayed below 95%. In multivariable logistic regression, data were compatible with no association between serostatus and gender conditional on age and country of origin (*p* = 0.60, Table 3). Moreover, no statistically significant association at the 5% level was found between serostatus and country of origin (Table 3).

**Table 3:** Multivariable logistic regression results investigating the association between serostatus and sociodemographic factors for measles, mumps, rubella, and varicella in homeless individuals in Germany (*n* = 585).

	**Adjusted OR**	**95% CI**		***P-*value**
**Serostatus Measles** ^a, b^				
**Age (years)**	1.06	1.04	1.09	< 0.0001
**Gender (Ref: Male)**				
Female	1.23	0.57	2.63	0.60
**Country of Origin (Ref: Germany)**				
EU-Origin	0.67	0.37	1.22	0.19
Non-EU-Origin	0.59	0.29	1.20	0.14
**Serostatus mumps** ^c^				
**Age (years)**	1.03	1.01	1.05	0.01
**Gender (Ref: Male)**				
Female	1.21	0.66	2.23	0.53
**Country of Origin (Ref: Germany)**				
EU-Origin	1.55	0.93	2.58	0.09
Non-EU-Origin	1.60	0.83	3.11	0.16
**Serostatus rubella** ^d^				
**Age (years)**	1.03	1.01	1.05	0.004
**Gender (Ref: Male)**				
Female	1.41	0.71	2.79	0.33
**Country of Origin (Ref: Germany)**				
EU-Origin	1.12	0.66	1.89	0.68
Non-EU-Origin	1.29	0.64	2.60	0.48
**Seroststatus varicella** ^e^				
**Age (years)**	1.01	0.98	1.05	0.43
**Gender (Ref: Male)**				
Female	0.61	0.23	1.59	0.31
**Country of Origin (Ref: Germany)**				
EU-Origin	0.81	0.33	1.98	0.65
Non-EU-Origin	0.88	0.27	2.85	0.84

^*a*^Hosmer-Lemshow test: p = 0.13. ^*b*^A polynomial functional form of age might provide improved model fit; no squared term for age was incorporated here aiming for a parsimonious model. ^*c*^Hosmer-Lemshow test: p = 0.35. ^*d*^Hosmer-Lemshow test: p = 0.98. ^*e*^Hosmer-Lemshow test: p = 0.48. CI, confidence interval; EU, European.

##### 3.2.1.1 Seroprevalence of measles in the homeless population according to the year of birth

**Figure 1:**
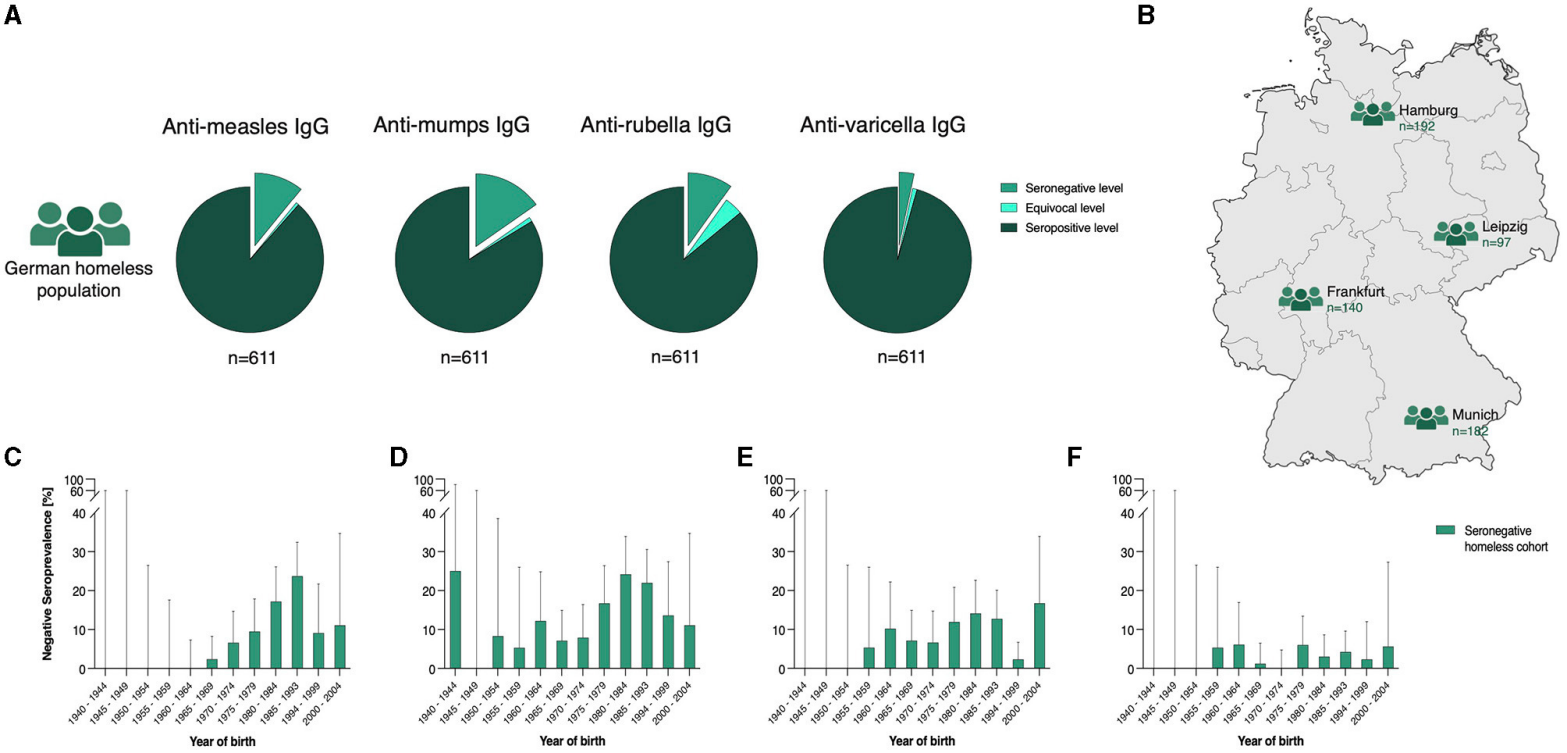
Seroprevalence of measles, mumps, rubella, and varicella in the German homeless population (green tones; *n* = 611 for measles, mumps, rubella, and varicella) is shown in **(A)**. Homeless individuals were enrolled from multiple centers all over Germany **(B)**. Seroprevalence of measles **(C)**, mumps **(D)**, rubella **(E)**, and varicella **(F)** (in %) are shown according to the year of birth in the German homeless population (green tones). Percentages of seronegative individuals are depicted. Exact 95% confidence intervals are depicted. GraphPad PRISM and Adobe Illustrator was used to create the figure.

Analyzing seroprevalence of measles in the context of the year of birth, the following observations were made: Neither seronegative nor equivocal antibody levels were discovered in homeless individuals born between 1940 and 1964. In homeless born between 1965 and 1993, the proportions of seronegative individuals increased with year of birth, reaching a peak of 23.7% (95% CI: 16.4–32.4) in individuals born between 1985 and 1993. In homeless individuals born after 1993, the proportion of seronegative individuals declined to 9-0.1 % (95% CI: 2.5–21.7) and 11.1% (95% CI: 1.4–34.7) (Figure 1 and Table 2). Consequently, seropositivity proportions stay below 95 % for individuals born 1970 or later when considering point estimates. When regarding the 95% CI, seropositivity proportions were not compatible with 95% for individuals born between 1980 and 1999. Data provided very strong evidence of an association between serostatus and age for measles conditional on gender and country of origin (*p* < 0.0001, Table 3). With every year increase in age, homeless individuals had 1.06 times the odds of seropositivity conditional on the covariates in the model (95% CI: 1.04–1.09) (Table 3).

#### 3.2.2 Seroprevalence of mumps in the homeless population

When considering the seroprevalence of mumps in the homeless population, 512/611 [83.8% (95% CI: 80.6–86.6)] homeless individuals were seropositive, 6/611 [1.0% (95% CI: 0.4–2.1)] had an equivocal level of antibodies, and 93/611 [15.2% (95% CI: 12.5–18.3)] were seronegative. 80/509 [15.7% (95% CI: 12.7–19.2)] of men and 13/101 [12.9% (95% CI: 7.0–21.0)] of women were seronegative for the mumps virus (Table 2), and the data were compatible with no association between serostatus and gender conditional on age and country of origin in multivariable logistic regression (*p* = 0.53; Table 3). Overall seropositivity proportions stayed below 84%. In addition, no statistically significant association was found between serostatus and country of origin as illustrated in Table 3.

##### 3.2.2.1 Seroprevalence of mumps in the homeless population according to the year of birth

Regarding the seroprevalence of mumps, we observed no seronegativity in homeless individuals born between 1945 and 1949. In homeless individuals born between 1950 and 1974, the proportions of seronegativity fluctuated between 5.3% (95% CI: 0.1–26.0) and 12.2% (95% CI: 4.6–24.8%). Higher proportions of seronegativity were observed in homeless adults born between 1975 and 1993, with most homeless individuals being seronegative if born between 1980 and 1984 [24.2% (95% CI: 16.2–33.9)]. Homeless individuals born after 1993 showed seronegativity proportions of 13.6% (95% CI: 5.2–27.4) and 11.1% (95% CI: 1.4–34.7). Consequently, seropositivity proportions stay below 92% for individuals born 1975 or later considering point estimates. When regarding the 95% CI, seropositivity proportions were not compatible with 92% for individuals born between 1975 and 1993. As for measles, data provided strong evidence of an association between serostatus and age conditional on gender and country of origin [aOR: 1.03 (95% CI: 1.01–1.05), *p* = 0.01, Table 3].

#### 3.2.3 Seroprevalence of rubella in the homeless population

When investigating the seroprevalence of rubella in the homeless cohort, 526/611 [86.1% (95% CI 83.1–88.7)] homeless individuals were seropositive, 25/611 [4.1% (95% CI: 2.7–6.0)] had an equivocal level antibody level, and 60/611 [9.8% (95% CI: 7.6–12.5)] were seronegative. 55/509 [10.8% (95% CI: 8.2–13.8)] men and 5/101 [5.0% (95% CI: 1.6–11.2)] women had a negative serostatus for the rubella virus. Data were compatible with no association between serostatus and gender conditional on age and the country of origin (*p* = 0.33; Table 3). No statistically significant association was found between serostatus and country of origin (Table 3).

##### 3.2.3.1 Seroprevalence of rubella in the homeless population according to the year of birth

We examined the association of rubella seroprevalence with the year of birth. Homeless individuals born between 1940 and 1954 showed no seronegativity. An overall upward trend regarding seronegativity proportions of homeless individuals can be observed in individuals born after 1955, ranging between 5.3% (95% CI: 0.1–26.0) and 14.1% (95% CI: 8.2 - 22.6). A dip in seronegativity [2.3% (95% CI: 0.1–12.0)] was found in homeless individuals born between 1994 and 1999. Yielding 16.7% (95% CI: 3.6–41.4), a peak in seronegativity proportions was observed in homeless individuals born between 2000 and 2004. Seropositivity proportions of below 83% were observed in individuals born between 1980 and 1993 as well as individuals born between 2000 and 2004, considering point estimates. When regarding the 95% CI, seropositivity proportions were not compatible with 83% for individuals born between 1985 and 1993. Data provided very strong evidence of an association between serostatus and age for rubella conditional on gender and country of origin (*p* = 0.004). With every year increase in age, homeless individuals had 1.03 times the odds of seropositivity conditional on the covariates in the model (95% CI: 1.01–1.05, Table 3).

#### 3.2.4 Seroprevalence of varicella in the homeless population

Lastly, when determining the seroprevalence of varicella in the homeless cohort, 585/611 [95.7% (95% CI: 93.8–97.2)] homeless individuals were tested seropositive, 6/611 [1.0% (95% CI: 0.4–2.1)] had an equivocal level of antibodies, and 20/611 [3.3% (95% CI: 2.0–5.0)] were seronegative. 16/509 [3.1% (95% CI: 1.8–5.1)] men and 4/101 [4.0% (95% CI: 1.1–9.8)] women were seronegative. Data were compatible with no association between serostatus and gender conditional on age and the country of origin in a multivariable logistic regression model (*p* = 0.31; Table 3). Additionally, no statistically significant association at the 5% level was found between serostatus and country of origin as demonstrated in Table 3.

##### 3.2.4.1 Seroprevalence of varicella in the homeless population according to the year of birth

We observed no seronegativity for varicella in individuals born between 1940 and 1954. The proportions of seronegativity slightly rise in homeless individuals born after 1955, fluctuating between 0.0% (95% CI: 0.0–4.7) and 6.1% (95% CI: 1.3–16.9) without revealing a clear pattern. Seropositivity proportions of above 93% were observed throughout all age groups. Data were compatible with no association between serostatus and age conditional on gender and the country of origin in multivariable logistic regression (*p* = 0.43, Table 3).

## 4 Discussion

So far, data on the seroprevalence of the vaccine-preventable diseases measles, mumps, rubella, and varicella in homeless individuals are missing. This study determined the seroprevalence of these viral diseases within the homeless cohort in Germany and found seronegativity proportions of 10.8% (95% CI: 8.5–13.5) for measles, 15.2% (95% CI: 12.5–18.3) for mumps, 9.8 % (95% CI: 7.6–12.5) for rubella, and 3.3% (95% CI: 2.0–5.0) for varicella.

One aim of the WHO, as well as of the German government, is the elimination of measles and rubella (46). A threshold of 95% is necessary to meet the WHO elimination goal for measles and rubella in Europe (47). To prevent virus circulation, seroprevalence proportions of > 95% and 83% to 86% were shown to suffice for measles and rubella, respectively (47, 48). In our sample of homeless individuals, the seroprevalence of measles was 88.5% (95% CI 85.8–91.0), and the seroprevalence of rubella was 86.1% (95% CI 83.1–88.7). While the seroprevalence of measles and rubella both fail to meet the targets of the WHO, the seroprevalence for rubella at least suffices for herd immunity in the homeless cohort (47). For mumps, a herd immunity threshold of 88 to 92% has been described (49, 50). However, in our sample of homeless individuals, only 83.8% (95% CI 80.6–86.6) were seropositive for mumps. The overall seroprevalence of varicella was 95.7% (95% CI 93.8–97.2), exceeding the 86%−91% threshold required for herd immunity against varicella, suggesting adequate immune protection for chickenpox (51). Consequently, herd immunity for rubella and varicella has been reached, while there is a persistent risk of transmission and spread of mumps and measles among homeless individuals in Germany.

In our study, the homeless gender was not statistically significantly associated with the serostatus of measles, mumps, rubella, and varicella. In line with that, seroprevalence data of the German general population also show no association between the serostatus of measles and mumps and gender. In contrast, data of the German general population described a statistically significant association between rubella serostatus and gender (42).

In total, 48.7% of our homeless individuals did not originate from Germany but immigrated from EU (32.8%) or non-EU (15.9%) countries. We did not find any statistically significant association between the serostatus of MMRV and the country of origin in this given cohort. This is interesting as different vaccination recommendations exist in different European countries. Moreover, the strength of national healthcare systems varies, with some countries lacking adequate healthcare structures or experiencing challenges such as the inability to provide access, adequate funding, or education for current immunization schedules (52). Residual confounding can be a reason for compatibility with the null hypothesis here, and stratifying by the national vaccination recommendation might better explain differences in MMRV seroprevalence between European countries. No evidence of a difference here, however, is in line with previous data where the seroprevalence of MMRV was determined in refugees coming to Germany in 2015. Here, a satisfactory proportion of protective immunity against MMRV was revealed with a seronegativity proportion of 7.4% (95% CI 5.5–9.6) for measles, 10.2% (95% CI 8.0–12.5) for mumps, 2.2% (95% CI 1.2–3.4) for rubella, and 3.3% (95% CI 1.9–4.9) for varicella (53).

If stratifying the serostatus by birth year, our data shows that seroprevalence of mumps, measles and rubella differed depending on the age of the homeless individual.

When regarding the 95% CI of the age-stratified seroprevalences for measles, seropositivity proportions were above 95% for individuals born before 1980 and drop below 95% after 1980, therefore not reaching the 95% herd immunity threshold for measles described in the literature. These results match the results of previous studies describing a decline in measles IgG after the implementation of measles vaccination in the 1970s and the broad usage of MMR combination vaccine in the 80s in Germany (42, 54). Higher proportions of seropositivity in homeless individuals born before implementation and broad usage may be explained by higher numbers of natural infections in these cohorts, yielding higher and more stable antibody levels (54). Nevertheless, studies also showed a high proportion of seropositivity even 20 years after the first MMR dose, particularly for rubella and measles (54). The decline in seronegativity proportions in individuals born after 1994 might be explained by the recommendation of a second measles vaccination in 1991 in Germany.

Mumps serostatus was associated with the age of homeless individuals without revealing a clear pattern. Yet, seronegativity proportions yielded higher in individuals born after 1974, peaked in individuals born between 1980 and 1993, and declined in individuals born after 1993. As for measles, introducing the mumps vaccine in the 70s may also have led to a decline in mumps IgG. With respect to the 95% confidence intervals of the age-stratified seroprevalences, herd immunity thresholds were not reached for individuals born between 1975 and 1993.

A declining number of natural childhood infections and, thus, immune protection prevalences in younger individuals emphasize the importance of targeted vaccination campaigns. Given that herd immunity thresholds are definitely not reached for individuals born after 1980 for measles and after 1975 for mumps, vaccination campaigns should particularly prioritize individuals within these age groups.

For rubella, an overall upward trend of seronegativity proportions was observed with decreasing age of the homeless individuals. Even though the herd immunity threshold is reached for rubella overall, when regarding the 95% confidence intervals of the age-stratified seroprevalences, herd immunity thresholds were not reached for individuals born between 1980 and 1993, indicating gaps in immune protection within these age groups.

Varicella serostatus, on the other hand, was not associated with the homeless individuals' age. Seropositivity proportions > 93% were discovered throughout all age groups, therefore unveiling a good immune protection status regardless of age. One explanation might be the late recommendation for the varicella vaccine in 2004 by the STIKO. As previously described for measles, it is possible that late vaccination recommendations led to high numbers of natural infections with varicella and high seroprevalence.

The German Health Interview and Examination Survey for adults (DESG1) is an interview and examination survey of the German adult population aged 18 years and older. It was conducted by the RKI from 2008 to 2011 to collect nationwide representative health data. In a cross-sectional analysis they determined the seroprevalence of MMR within the German general population.

When comparing their data of the German general population with our data of the homeless population, we see that overall, proportions of seronegativity for measles, mumps, and rubella were higher in homeless individuals than in the general population (10.8% vs. 6.4% for measles, 15.2% vs. 10.3% for mumps, 9.8 % for rubella vs. 4.4% for rubella). In addition, seronegativity proportions appeared to be higher in homeless men than in men of the general population in Germany. No considerable differences were observed between seroprevalence data of homeless women and women of the general population.

However, no differences were observed when comparing MMR seroprevalences in different age groups of the homeless and general population cohorts. Both the German general population and homeless cohort showed age-dependent fluctuations regarding MMR seroprevalence, as both may have been equally affected by the rollout of new vaccines. MMR is an infection and recommended vaccination during childhood. Given the short duration of homelessness observed in our cohort, homeless individuals are likely to have the same exposure history as the general population (likely not to have been homeless during childhood).

Differences in the overall seroprevalence between the two cohorts might thus mainly arise from the different age structures of the two cohorts. Specifically, our study included a higher proportion of younger individuals, who likely exhibit higher seronegativity proportions, while Friedrich et al. (42) included more individuals of older age groups, likely exhibiting lower seronegativity proportions. It is important to acknowledge that the aforementioned comparisons might suffer statistical power to detect true differences in MMRV seroprevalences – if any – between the German general and homeless population and that further research is needed to address this question.

During the COVID-19 pandemic, it was only through protection measures that the incidence of measles was < 1 per 1,000,000 inhabitants for the first time; the WHO's target was missed by a wide margin (55). The increase in measles incidence rates, as a consequence of the repeal of COVID-19-related measures and an accompanying decrease in vaccination coverage in recent years, urgently requires targeted interventions to prevent the disease's further spread and accomplish the WHO elimination goal (23). Marginalized groups, such as people experiencing homelessness, are considered particularly important transmitters and susceptible to adverse outcomes for infectious diseases due to crowded living conditions, low hygiene standards, and high somatic and psychological morbidity (5). Against this background, targeted elimination campaigns should include and prioritize homeless individuals. Neglecting the homeless could lead to recurrent infections in these vulnerable groups and potentially beyond.

Various strategies have been proposed in the literature to increase vaccination uptake in the homeless population. One such strategy is to offer on-site vaccination services at community facilities for the homeless. This can help to overcome the barriers to medical help that homeless individuals often face by providing immediate access to vaccinations. Collaboration between healthcare providers and homeless service providers can also facilitate the delivery and distribution of vaccines. Education programs that address the benefits and potential concerns of vaccination may also be effective in increasing vaccination uptake among the homeless (56).

### 4.1 Strengths and limitations

Until now, there was no data on the seroprevalence of MMRV in homeless individuals in Europe. This study is the first to investigate immune protection levels against these vaccine-preventable diseases, aiming to reveal potential gaps in immunization. Moreover, it helps unveil key predictors of seronegativity and, thus, populations at increased risk of infection (57, 58). While those studies provide a good measure of immune protection, they tend to underestimate a population's immunity as IgG antibody levels wane with time and might not be detectable despite vaccination or past infection (57). Contrary to this criticism, previous studies reveal high antibody levels, particularly for measles and mumps, even 20 years after vaccination (54). Moreover, vaccination coverage data, on the other hand, gathered with the help of reports and surveys, does not always correctly represent a population's immunity due to difficulties in completely and accurately acquiring such data (59, 60). We did not consider cell-derived immunity here, which also contributes to protecting against these viral diseases (61). The homeless population, being highly mobile, is challenging to reach. However, this study is the first and largest of its kind, examining the seroprevalence of MMRV in homeless individuals in Germany and beyond. In our study, we only included individuals who use social services. Consequently, our data is representative only of homeless individuals using such services. We used the ETHOS to account for potential differences due to the accommodation status. Unfortunately, the proportion of women within the overall study sample was relatively small, a common problem in previous studies of the homeless population (62). Further research is needed to investigate differences in MMR seroprevalence between the German general and homeless population, as our comparison might lack sufficient power to detect any true differences given the small sample size in some age groups of the homeless cohort. Moreover, a comparison of 95% confidence intervals is a conservative approach with no statistical tests done where primary data were not available. Lastly, differences in the age structures limit study comparability.

### 4.2 Conclusions

In conclusion, overall herd immunity thresholds were not achieved for measles and mumps, but a satisfactory overall immunization status for rubella and varicella was observed in the homeless cohort. Likewise, the WHO elimination goals for measles and rubella were not achieved. Roughly every 10th and approximately every 7th homeless individual has no immune protection against measles and mumps. Age was identified as an important predictor of seronegativity for MMR in homeless individuals, which aligns with data from the German general population (42). Vaccination campaigns should particularly address homeless individuals born after 1980 and 1975 for measles and mumps, respectively, as herd immunity thresholds are not reached for those age groups. As homeless individuals are especially vulnerable and have high transmission rates, therefore, targeted elimination strategies and campaigns are critical.

## Data availability statement

The datasets presented in this article are not readily available because of ethical restrictions involving patient data but may be available from the corresponding author upon reasonable request. Requests to access the datasets should be directed to fa.heinrich@uke.de.

## Ethics statement

The study was conducted in accordance with the declaration of Helsinki. Ethical approval was obtained from the Ethics Committee of the Hamburg Chamber of Physicians (application number: PV7333). The participants provided their written informed consent to participate in this study.

## Author contributions

WG: Methodology, Formal analysis, Writing—original draft, Writing—review & editing. FB: Conceptualization, Writing—review & editing, Funding acquisition. KD: Writing—review & editing. AB: Writing—review & editing. VK: Writing—review & editing. VR: Writing—review & editing. DN: Writing—review & editing. BW: Writing—review & editing. BO: Writing—review & editing, Supervision. KP: Conceptualization, Writing—review & editing, Funding acquisition, Supervision. SP: Writing—review & editing, ML: Writing—review & editing. FH: Conceptualization, Methodology, Formal analysis, Writing—original draft, Writing—review & editing, Funding acquisition, Supervision.

## References

[1] Wegener D. Steigende Zahl Wohnungsloser Im Wohnungslosensektor, Wohnungslosigkeit Anerkannter Geflüchteter Sinkt BfW e.V. (2021) Available online at: https://www.bagw.de/de/presse/show?tx_netnews_newsview%5Baction%5D=show&tx_netnews_newsview%5Bcontroller%5D=News&tx_netnews_newsview%5Bnews%5D=215&cHash=27c6924963ddf518fabd193bd1a14f56 (accessed April 28, 2023).

[2] Ly TDA Castaneda S Hoang VT Dao TL Gautret P. Vaccine-preventable diseases other than tuberculosis, and homelessness: a scoping review of the published literature, 1980 to 2020. Vaccine. (2021) 39:1205–24. 10.1016/j.vaccine.2021.01.035 33509694

[3] Peak CM Stous SS Healy JM Hofmeister MG Lin Y Ramachandran S. Homelessness and hepatitis a-San Diego County, 2016-2018. Clin Infect Dis. (2020) 71:14–21. 10.1093/cid/ciz788 31412358 PMC10956402

[4] Noska AJ Belperio PS Loomis TP O'Toole TP Backus LI. Prevalence of human immunodeficiency virus, hepatitis C virus, and hepatitis B virus among homeless and nonhomeless United States veterans. Clin Infect Dis. (2017) 65:252–8. 10.1093/cid/cix295 28379316 PMC6248754

[5] Raoult D Foucault C Brouqui P. Infections in the homeless. Lancet Infect Dis. (2001) 1:77–84. 10.1016/S1473-3099(01)00062-7 11871479

[6] Hwang SW Kiss A Ho MM Leung CS Gundlapalli AV. Infectious disease exposures and contact tracing in homeless shelters. J Health Care Poor Underserved. (2008) 19:1163–7. 10.1353/hpu.0.0070 19029743 PMC4465825

[7] Schindel D Kleyer C Schenk L. [Somatic diseases of homeless people in Germany. A narrative literature review for the Years 2009-2019]. Bundesgesundheitsblatt Gesundheitsforschung Gesundheitsschutz. (2020) 63:1189–202. 10.1007/s00103-020-03213-9 32940746

[8] Schreiter S Speerforck S Schomerus G Gutwinski S. Homelessness: care for the most vulnerable - a narrative review of risk factors, health needs, stigma, and intervention strategies. Curr Opin Psychiatry. (2021) 34:400–4. 10.1097/YCO.0000000000000715 33993170

[9] Salize HJ Dillmann-Lange C Stern G Kentner-Figura B Stamm K Rössler W. Alcoholism and somatic comorbidity among homeless people in Mannheim, Germany. Addiction. (2002) 97:1593–600. 10.1046/j.1360-0443.2002.00235.x 12472643

[10] Langnäse K Müller MJ. Nutrition and health in an adult urban homeless population in Germany. Public Health Nutr. (2001) 4:805–11. 10.1079/PHN2000119 11415488

[11] Nielsen SF Hjorthøj CR Erlangsen A Nordentoft M. Psychiatric disorders and mortality among people in homeless shelters in denmark: a nationwide register-based cohort study. Lancet. (2011) 377:2205–14. 10.1016/S0140-6736(11)60747-2 21676456

[12] Nilsson SF Nordentoft M Fazel S Laursen TM. Homelessness and police-recorded crime victimisation: a nationwide, register-based cohort study. The Lancet Public Health. (2020) 5:e333–e41. 10.1016/S2468-2667(20)30075-X 32504586 PMC7618086

[13] Nusselder WJ Slockers MT Krol L Slockers CT Looman CW van Beeck EF. Mortality and life expectancy in homeless men and women in Rotterdam: 2001-2010. PLoS One. (2013) 8:e73979. 10.1371/journal.pone.0073979 24098329 PMC3788767

[14] Fazel S Geddes JR Kushel M. The health of homeless people in high-income countries: descriptive epidemiology, health consequences, and clinical and policy recommendations. Lancet. (2014) 384:1529–40. 10.1016/S0140-6736(14)61132-6 25390578 PMC4520328

[15] Ramsay N Hossain R Moore M Milo M Brown A. Health care while homeless: barriers, facilitators, and the lived experiences of homeless individuals accessing health care in a Canadian regional municipality. Qual Health Res. (2019) 29:1839–49. 10.1177/1049732319829434 30810072

[16] Davies A Wood LJ. Homeless health care: meeting the challenges of providing primary care. Med J Aust. (2018) 209:230–4. 10.5694/mja17.01264 30157413

[17] Kaduszkiewicz H Bochon B van den Bussche H Hansmann-Wiest J van der Leeden C. The medical treatment of homeless people. Dtsch Arztebl Int. (2017) 114:673–9. 10.3238/arztebl.2017.0673 29070427 PMC5963585

[18] Robert-Koch-Institut. Nationales Referenzzentrum Für Masern, Mumps, Röteln. Available online at: https://www.rki.de/DE/Content/Infekt/NRZ/MMR/mmr_node.html (accessed November 18, 2023).

[19] Moss WJ. Measles. Lancet. (2017) 390:2490–502. 10.1016/S0140-6736(17)31463-0 28673424

[20] Hviid A Rubin S Mühlemann K. Mumps. Lancet. (2008) 371:932–44. 10.1016/S0140-6736(08)60419-5 18342688

[21] Lambert N Strebel P Orenstein W Icenogle J Poland GA. Rubella. Lancet. (2015) 385:2297–307. 10.1016/S0140-6736(14)60539-0 25576992 PMC4514442

[22] Freer G Pistello M. Varicella-zoster virus infection: natural history, clinical manifestations, immunity and current and future vaccination strategies. New Microbiol. (2018) 41:95–105. 29498740

[23] European Centre for Disease Prevention and Control. Measles. In: Annual Epidemiological Report for 2022. Stockholm: ECDC (2023). Available online at: https://www.ecdc.europa.eu/sites/default/files/documents/Measles%20Annual%20Epidemiological%20Report%202022%20data.pdf

[24] European Centre for Disease Prevention and Control. Measles. In: Annual Epidemiological Report for 2018. Stockholm: ECDC (2020). Available online at: https://www.ecdc.europa.eu/sites/default/files/documents/measles-annual-epidemiologicalreport-2018.pdf

[25] Ärzteblatt. Mehr Masernfälle in Deutschland (2024). Available online at: https://www.aerzteblatt.de/nachrichten/149206/Mehr-Masernfaelle-in-Deutschland#:~:text=In%20Deutschland%20wurden%20laut%20STIKO,waren%20es%2080%20Fälle%20gewesen (accessed March 6, 2024).

[26] World-Health-Organization. 30-Facher Anstieg Der Masernfälle Im Jahr 2023 Sorgt Für Dringenden Handlungsbedarf in Der Europäischen Region Der Who (2023). Available online at: https://www.who.int/europe/de/news/item/14-12-2023-a-30-fold-rise-of-measles-cases-in-2023-in-the-who-european-region-warrants-urgent-action (accessed December 14, 2023).

[27] Robert-Koch-Institut. Epidemiologische Situation Der Masern Und Röteln in Deutschland in 2022. (2023) Available online at: https://www.rki.de/DE/Content/Infekt/Impfen/Praevention/elimination_04_01.html#:~:text=Vom%201.,Fall%20pro%201%20Million%20Einwohner (accessed March 6, 2024).

[28] European Centre for Disease Prevention and Control. Measles and Rubella Surveillance - 2017. Stockholm: ECDC (2018). Available online at: https://www.ecdc.europa.eu/sites/default/files/documents/Measles-and-Rubella-Surveillance-2017.pdf

[29] Robert-Koch-Institut. Survstat@Rki 2.0. (2024) Available online at: https://survstat.rki.de/Content/Query/Create.aspx (accessed March 12, 2024).

[30] Robert-Koch-Institut. Rki-Ratgeber Mumps (2023). Available online at: https://www.rki.de/DE/Content/Infekt/EpidBull/Merkblaetter/Ratgeber_Mumps.html#:~:text=Seit%20Einführung%20der%20Meldepflicht%20im,6%20Erkrankungen%20pro%20100.000%20Einwohner (accessed March 6, 2024).

[31] European Centre for Disease Prevention and Control. Varicella Vaccination in the European Union. Stockholm: ECDC (2015). Available online at: https://www.ecdc.europa.eu/sites/default/files/media/en/publications/Publications/Varicella-Guidance-2015.pdf

[32] Robert-Koch-Institut. Stiko-Empfehlungen – Historie. (2018). Available online at: https://www.rki.de/DE/Content/Infekt/Impfen/Materialien/Poster/Poster_Stiko_Hist.pdf?__blob=publicationFile (accessed September 4, 2022).

[33] Deutsche-Apotheker-Zeitung. Vierfachimpfstoff: Erster Mmrv-Impfstoff Zugelassen. (2006) Available online at: https://www.deutsche-apotheker-zeitung.de/daz-az/2006/daz-33-2006/uid-16347 (accessed September 4, 2022).

[34] Robert-Koch-Institut. Epidemiologisches Bulletin - Masern in Deutschland Und Weltweit. (2022).

[35] Robert-Koch-Institut. Masernschutzgesetz. (2022) Available online at: https://www.rki.de/DE/Content/Infekt/Impfen/ImpfungenAZ/MMR_Masern/Masernschutzgesetz.html (accessed March 5, 2024).

[36] Bundesministerium-für-Gesundheit. Impfpflicht Soll Kinder Vor Masern Schützen. Available online at: https://www.bundesgesundheitsministerium.de/impfpflicht.html (accessed September 4, 2022).

[37] European-Centre-for-Disease-Prevention-and-Control. Varicella: Recommended Vaccinations. (2024) Available online at: https://vaccine-schedule.ecdc.europa.eu/Scheduler/ByDisease?SelectedDiseaseId=11&SelectedCountryIdByDisease=-1 (accessed March 12, 2024).

[38] European-Centre-for-Disease-Prevention-and-Control. Mumps: Recommended Vaccinations. (2024) Available online at: https://vaccine-schedule.ecdc.europa.eu/Scheduler/ByDisease?SelectedDiseaseId=8&SelectedCountryIdByDisease=-1 (accessed March 12, 2024).

[39] European-Centre-for-Disease-Prevention-and-Control. Rubella: Recommended Vaccinations. (2024) Available online at: https://vaccine-schedule.ecdc.europa.eu/Scheduler/ByDisease?SelectedDiseaseId=10&SelectedCountryIdByDisease=-1 (accessed March 12, 2024).

[40] European-Centre-for-Disease-Prevention-and-Control. Measles: Recommended Vaccinations. (2024) Available online at: https://vaccine-schedule.ecdc.europa.eu/Scheduler/ByDisease?SelectedDiseaseId=8&SelectedCountryIdByDisease=-1 (accessed March 12, 2024).

[41] Robert-Koch-Institut. Impfquoten (2022) Available online at: https://www.rki.de/DE/Content/Infekt/Impfen/Impfstatus/impfstatus_node.html (accessed September 4, 2024).

[42] Friedrich N Poethko-Müller C Kuhnert R Matysiak-Klose D Koch J Wichmann O. Seroprevalence of measles-, mumps-, and rubella-specific antibodies in the german adult population - cross-sectional analysis of the German health interview and examination survey for adults (Degs1). Lancet Reg Health Eur. (2021) 7:100128. 10.1016/j.lanepe.2021.100128 34557838 PMC8454806

[43] Washington-Brown L Cirilo RW. Advancing the health of homeless populations through vaccinations. J Am Assoc Nurse Pract. (2020) 33:824–30. 10.1097/JXX.0000000000000509 33038117

[44] European-Federation-of-National-Associations-Working-with-the-Homeless-AISBL. Ethos – EuropäIsche Typologie FüR Obdachlosigkeit, Wohnungslosigkeit Und PrekäRe Wohnversorgung. (2024) Available online at: https://www.feantsa.org/download/at___6864666519241181714.pdf (accessed March 6, 2024).

[45] Bertram F Hajek A Dost K Graf W Brennecke A Kowalski V. The mental and physical health of the homeless. Dtsch Arztebl Int. (2022) 119:861–8. 10.3238/arztebl.m2022.0357 36382585 PMC9989961

[46] Robert-Koch-Institut. Elimination Der Masern Und Röteln in Deutschland. (2022). Available online at: https://www.rki.de/DE/Content/Infekt/Impfen/Praevention/elimination_04.html/with (accessed August 25, 2022).

[47] O'Connor P Jankovic D Zimmerman L Ben Mamou M Reef S. Progress toward rubella elimination - world health organization European region, 2005-2019. MMWR Morb Mortal Wkly Rep. (2021) 70:833–9. 10.15585/mmwr.mm7023a1 34111057 PMC8191869

[48] World-Health-Organization. Eliminating Measles and Rubella. Framework for the Verification Process in the Who European Region. Geneva: WHO (2014).

[49] Anderson RM May RM. Vaccination and herd immunity to infectious diseases. Nature. (1985) 318:323–9. 10.1038/318323a0 3906406

[50] Fine PE. Herd immunity: history, theory, practice. Epidemiol Rev. (1993) 15:265–302. 10.1093/oxfordjournals.epirev.a036121 8174658

[51] Plans-Rubió P. Evaluation of the establishment of herd immunity in the population by means of serological surveys and vaccination coverage. Hum Vaccin Immunother. (2012) 8:184–8. 10.4161/hv.18444 22426372

[52] Hardt K Bonanni P King S Santos JI El-Hodhod M Zimet GD. Vaccine strategies: optimising outcomes. Vaccine. (2016) 34:6691–9. 10.1016/j.vaccine.2016.10.078 27887796

[53] Jablonka A Happle C Grote U Schleenvoigt BT Hampel A Dopfer C. Measles, Mumps, Rubella, and Varicella Seroprevalence in Refugees in Germany in 2015. Infection. (2016) 44:781–7. 10.1007/s15010-016-0926-7 27449329

[54] Davidkin I Jokinen S Broman M Leinikki P Peltola H. Persistence of Measles, Mumps, and Rubella Antibodies in an Mmr-vaccinated cohort: a 20-year follow-up. J Infect Dis. (2008) 197:950–6. 10.1086/528993 18419470

[55] Robert-Koch-Institut. Aktuelles Zu Masern in Deutschland Und Weltweit Epidemiologisches Bulletin. (2022) Available online at: https://www.rki.de/DE/Content/Infekt/EpidBull/Archiv/2022/Ausgaben/34_22.pdf?__blob=publicationFile (accessed August 22, 2022).

[56] McCosker LK El-Heneidy A Seale H Ware RS Downes MJ. Strategies to improve vaccination rates in people who are homeless: a systematic review. Vaccine. (2022). 10.1016/j.vaccine.2022.04.022 35484042 PMC9040475

[57] Cutts FT Hanson M. Seroepidemiology: an underused tool for designing and monitoring vaccination programmes in low- and middle-income countries. Trop Med Int Health. (2016) 21:1086–98. 10.1111/tmi.12737 27300255

[58] Wilson SE Deeks SL Hatchette TF Crowcroft NS. The role of seroepidemiology in the comprehensive surveillance of vaccine-preventable diseases. CMAJ. (2012) 184:E70–6. 10.1503/cmaj.110506 22083674 PMC3255197

[59] Cutts FT Izurieta HS Rhoda DA. Measuring coverage in MNCH: design, implementation, and interpretation challenges associated with tracking vaccination coverage using household surveys. PLoS Med. (2013) 10:e1001404. 10.1371/journal.pmed.1001404 23667334 PMC3646208

[60] Murray CJ Shengelia B Gupta N Moussavi S Tandon A Thieren M. Validity of reported vaccination coverage in 45 countries. Lancet. (2003) 362:1022–7. 10.1016/S0140-6736(03)14411-X 14522532

[61] Robert-Koch-Institut. Labor, Diagnostik, Kosten Und Dokumentation (2021). Available online at: https://www.rki.de/SharedDocs/FAQ/MMR/Masernimmunitaet/Liste_Masernimmunitaet.html (accessed August 28, 2022).

[62] van Rüth V König HH Bertram F Schmiedel P Ondruschka B Püschel K. Determinants of health-related quality of life among homeless individuals during the COVID-19 pandemic. Public Health. (2021) 194:60–6. 10.1016/j.puhe.2021.02.026 33865148

