# Supplementary material for: Immunity against measles, mumps, rubella, and varicella among homeless individuals in Germany — A nationwide multi-center cross-sectional study
Source: Front Public Health. 2024 May 9;12:1375151. doi: 10.3389/fpubh.2024.1375151 (PMC11111963; doi:10.3389/fpubh.2024.1375151)
Supplement: Supplementary file 1 [file Data_Sheet_1.PDF]

Supplementary Table 1. Sample characteristics of homeless individuals with (n=611) and without (n=60) seroprevalence data.

	**Homeless individuals without seroprevalence data** Median (IQR)/ number (%)	**Homeless individuals with seroprevalence data** Median (IQR)/ number (%)	**Homeless individuals with and without seroprevalence data** Median (IQR)/ number (%)
	n=60	n=611	n=671
**Gender**			
Male	19 (55.9%)	509 (83.4%)	528 (82.0%)
Female	15 (44.1%)	101 (16.6%)	116 (18.0%)
**Age** (years)	41 (37 - 51)	43 (35 - 53)	43 (35 - 52)
**Country of origin**			
Germany	20 (58.8%)	301 (51.4%)	321 (51.8%)
EU-country	8 (23.5%)	192 (32.8%)	200 (32.3%)
Non-EU-country	6 (17.6%)	93 (15.9%)	99 (16.0%)
**Education**			
No degree	7 (20.6%)	106 (18.1%)	113 (18.3%)
School education	14 (41.2%)	272 (46.5%)	286 (46.2%)
Vocational education	11 (32.4%)	163 (27.9%)	174 (28.1%)
Higher tertiary education	2 (5.9%)	44 (7.5%)	46 (7.4%)
**Marital status**			
Married and married living apart	5 (15.2%)	69 (11.8%)	74 (11.9%)
Single	22 (66.7%)	394 (67.0%)	416 (67.0%)
Widowed	1 (3.0%)	15 (2.6%)	16 (2.6%)
Divorced	5 (15.2%)	110 (18.7%)	115 (18.5%)
**Occupation**	5 (15.6%)	66 (11.7%)	71 (11.9%)
**On welfare**	21 (61.8%)	266 (45.2%)	287 (46.1%)
**Health Insurance**	26 (78.8%)	400 (67.0%)	426 (67.6%)
**Duration of homelessness** (months)	24 (5 - 60)	18 (6 - 48)	18 (6 - 48)
**ETHOS**			
Living Rough	11 (34.4%)	238 (41.3%)	249 (41.0%)
Emergency accommodation	7 (21.9%)	92 (16.0%)	99 (16.3%)
Accommodation for the homeless	14 (43.8%)	210 (36.5%)	224 (36.8%)
Women's Shelter	0 (0.0%)	30 (5.2%)	30 (4.9%)
Accommodation for immigrants	0 (0.0%)	3 (0.5%)	0 (0.5%)
People in long term accommodation	0 (0.0%)	3 (0.5%)	0 (0.5%)

Numbers and percentages of non-missing observations are depicted. Abbreviations: EU, European; ETHOS, European Typology of Homelessness.
